# Balancing Fairness and Efficiency: The Impact of Identity-Blind and Identity-Conscious Accountability on Applicant Screening

**DOI:** 10.1371/journal.pone.0145208

**Published:** 2015-12-14

**Authors:** William T. Self, Gregory Mitchell, Barbara A. Mellers, Philip E. Tetlock, J. Angus D. Hildreth

**Affiliations:** 1 Henry W. Bloch School of Management, University of Missouri-Kansas City, Kansas City, Missouri, United States of America; 2 School of Law, University of Virginia, Charlottesville, Virginia, United States of America; 3 Department of Psychology, University of Pennsylvania, Philadelphia, Pennsylvania, United States of America; 4 Haas School of Business, University of California, Berkeley, California, United States of America; Georgetown University Medical Center, UNITED STATES

## Abstract

This study compared two forms of accountability that can be used to promote diversity and fairness in personnel selections: identity-conscious accountability (holding decision makers accountable for which groups are selected) versus identity-blind accountability (holding decision makers accountable for making fair selections). In a simulated application screening process, undergraduate participants (majority female) sorted applicants under conditions of identity-conscious accountability, identity-blind accountability, or no accountability for an applicant pool in which white males either did or did not have a human capital advantage. Under identity-conscious accountability, participants exhibited pro-female and pro-minority bias, particularly in the white-male-advantage applicant pool. Under identity-blind accountability, participants exhibited no biases and candidate qualifications dominated interview recommendations. Participants exhibited greater resentment toward management under identity-conscious accountability.

## Introduction

Traditionally organizations have sought to ensure equal treatment of employees and applicants by focusing personnel managers on each individual’s attributes and performance and proscribing consideration of group membership in performance reviews and selection decisions [1]. This colorblind, or more broadly “identity blind,” approach [2] to personnel management is attractive to organizations because it avoids a tradeoff between fairness and efficiency: by avoiding group-based discrimination, stronger candidates will be selected over weaker candidates regardless of their sex, race or ethnicity, thereby creating a more capable and efficient workforce.

Given persistent inequalities in representation and pay for women and minorities, however, the effectiveness of an identity-blind approach to personnel management has been called into question. Some argue that implicit biases operating beyond conscious control render instructions to treat people equally and provide insufficient protection against discrimination (e.g., [3]). Others argue that belief in the effectiveness of an identity-blind approach to personnel management entrenches disparities and protects white, male advantages in organizations (e.g., [4]). From this perspective, organizations must empower high-level officers to monitor and redress disparities in the workforce through affirmative actions aimed at overcoming white, male advantages in hiring, promotion and pay (e.g., [5, 6]). This identity-conscious approach to personnel management emphasizes holding managers accountable for achieving specified representation goals rather than following fair procedures (e.g., [7, 8]).

An important question raised by the identity-conscious approach to personnel management is whether organizations using this approach can increase diversity without compromising efficiency and without encouraging reverse discrimination against white males. In short, do identity-conscious policies work better than identity-blind policies at ensuring that talented minorities and women are selected, or do identity-conscious policies lead managers to place group identity over individual skills and abilities? The present study examined these questions at the initial, applicant screening stage of the personnel management process, examining how screeners behave when they know they will be held accountable for following a fair selection process versus how they behave when they know they will be held accountable for increasing the diversity of the workforce.

### Accountability in Personnel Management

The screening of applicants for interviews constitutes the first and, for those who do not progress, the most important step in an organization’s personnel selection process. Permitting group biases to influence selections at this step in the hiring process increases the likelihood of litigation and decreases the likelihood that the best candidates will be interviewed and ultimately selected. Accordingly, personnel psychologists have devoted considerable effort to identifying conditions under which candidate qualifications, rather than candidate race, gender or other inappropriate factors, drive interview selections (e.g., [9–12]).

One of the most promising avenues for debiasing the interview selection process involves holding selectors accountable for their decisions. Numerous studies have found that those who expect to have to explain their decisions to another make more accurate and less biased decisions (e.g., [13–17]). However, not all studies find positive accountability effects. For instance, in two studies Gordon, Rozelle and Baxter [18, 19] found that accountable raters exhibited greater age bias than unaccountable raters in their candidate evaluations.

One explanation for the differential effects of accountability involves participant expectations about which decisions will be seen as a defensible or desirable to the audience. Particularly relevant to employment settings are studies that find that raters who are accountable to ratees tend to inflate their evaluations relative to raters who are accountable to superiors (e.g., [20–22]). For instance, Mero, Guidice and Brownlee [23] found that raters who were accountable to ratees inflated their performance ratings by emphasizing the positive aspects of the ratee’s performance, whereas raters accountable to a graduate student administering the study took into account the full range of the ratee’s performance and provided more accurate ratings. Thus, a decision-maker’s expectations about the audience have important implications for which decisions will be made and for what supporting evidence will be seen as defensible [24]. Also, different accountability instructions can lead decision-makers to attend to different evidence or to pursue different goals. Gordon, Rozelle and Baxter [18, 19], for instance, imposed an accountability manipulation that focused their participants on remembering details about particular candidates, a manipulation that these researchers hypothesized would make stereotype-consistent information more memorable and thus more influential in evaluations, leading to greater age bias.

Audience effects are much studied within the accountability literature, but the effects of different accountability instructions have been much less studied. In many accountability studies, those being monitored are told simply that they will be asked to disclose or explain their decisions to one or more persons with no particular goal or process specified (see, e.g., [14, 21]). In the personnel selection context, however, organizations often give their managers more specific accountability instructions aimed at promoting the organization’s selection goals. In particular, depending on the external legal climate and internal commitments to diversity, an organization is likely to impose either an identity-blind or identity-conscious set of accountability instructions on those making selection decisions (see, e.g., [1, 2, 25, 26]).

With identity-blind (IB) accountability, personnel managers are told that they will be held accountable for making decisions fairly and consistently, based on job-relevant considerations. This form of selection accountability is common among American and European organizations, as it tracks the default legal directive that firms’ personnel selection processes provide equal opportunity to applicants regardless of their race, sex, age or other legally protected characteristics (e.g., [27,28]). With identity-conscious (IC) accountability, an organization encourages its personnel managers to take affirmative steps to ensure that women and minorities are given full consideration and encourages managers to take steps to try to increase the representation of these groups within the workforce. This form of accountability is likely to be found among organizations facing legal pressure or pressure from advocacy groups to rectify disparities in minority or female representation [5, 29].

No prior studies have examined the impact of these two contrasting forms of accountability on personnel selections. Previous studies have compared process and outcome accountability (i.e., have compared the effects of focusing participants on *how* a decision is made versus *what* decision is made), but the conceptions of accountability used in these studies do not correspond to IB and IC selection accountability. Most instructive for the employment context is Brtek and Motowidlo [13], in which participants viewed 30 taped interviews and evaluated responses to one of the questions answered by interviewees under conditions of no accountability, procedural accountability (participants were told they would meet with experimenters to justify the procedure they used to evaluate interviewees), outcome accountability (participants were told their ratings would be compared to expert ratings of the same interviews), or both procedural and outcome accountability. Brtek and Motowidlo [13] found that their procedural accountability manipulation increased the validity of participants’ interview ratings (as judged by comparison to supervisor ratings of performance), but their outcome accountability manipulation decreased the validity of participants’ ratings. Under the procedural accountability instruction, participants were more attentive to interview information, which led to ratings with greater validity.

A number of studies outside the employment context have contrasted process and outcome accountability (e.g., [30–31]). Pitesa and Thau [32], for example, examined process and outcome accountability effects on self-serving behavior by agents making simulated financial investment decisions, with process accountability operationalized as the expectation that the decision-making process would have to be explained to the experimenter and outcome accountability operationalized as the expectation that the expected outcome from that choice would have to be explained. They found that outcome accountability increased self-serving investment choices (i.e., risky choices that were likely to provide a profit to the agent but result in a loss for the principal), while process accountability decreased self-serving behavior by reducing the agent’s subjective sense of power to act.

Although the process accountability manipulations used in these studies approximate IB accountability as found in the personnel selection context, those forms of accountability do not expressly prescribe that decisions be based on job-relevant information and be blind to group membership, and the outcome accountability manipulations in these studies do not approximate IC accountability of the kind found when companies implement affirmative action initiatives. Accordingly, none of the prior studies directly addresses how managers make selection decisions under conditions of IB and IC accountability.

### Contrasting Identity-Blind and Identity-Conscious Selection Accountability

Ideally, IB and IC accountability would both prevent the expression of bias against women and minorities without triggering a counter-bias against males and whites. Some social scientists fear, however, that IB accountability is too weak to combat the conscious and unconscious biases that can infect selection processes, particularly where evidence of job qualifications is ambiguous and personnel managers have discretion to weigh the ambiguous evidence and apply subjectively-determined standards of merit (e.g., [33–34]). Under these conditions, wily selectors can shift their standards of evidence or conception of merit as needed to conceal their discriminatory behavior [35]. Even well-intentioned managers who believe they are trying to follow a fair process may fail to appreciate how stereotypes and shifting standards of merit affect their decisions, or they may over-correct for the hard-to-detect biases they fear they possess [36]. From this view, rigorously applied IC accountability is the only sure protection against gender- or race-based disparities in personnel selection (e.g., [2, 37, 38]).

The fear about IC accountability is that it is too strong a medicine for the ill it seeks to cure. Even where IC accountability instructions are designed to avoid hard quotas, selectors may implicitly adopt numerical goals to satisfy accountability demands [39] or may devote too much attention to candidates from the high-interest groups and too little attention to candidates from the low-interest groups. The risk is that IC accountability becomes a special case of accountability to an audience with known views that motivates decision-makers to conform without having positive effects on the rigor of the underlying judgment-and-choice processes [40]. In this view, although representation gains may be made through IC accountability, these gains come at the cost of decreased focus on job-relevant qualifications in selections [41] and increased exposure to claims of reverse discrimination [42], perceptions of distributive and procedural injustice [43–44], stereotyping of, and self-doubt among, beneficiaries of the identity-conscious measures [45–47], and backlash against diversity initiatives and remedial policies [48–49].

The impact that each form of accountability has on the selection process will likely depend on the make-up of the labor pool. In labor markets where the target groups suffer from relative human capital deficits due to a history of discrimination, IB accountability should prevent discrimination based on sex or race but allow any existing disparities within workforces to persist, whereas IC accountability may lead decision-makers to place inclusion above qualifications. In general, when the tradeoff between group membership and job qualifications is zero, and there is no correlation between group membership and job qualifications, the undesired side-effects of identity-conscious accountability should be minimal. But as the correlation becomes increasingly negative (i.e., as disparities in human capital between groups grow), reverse discrimination should become more difficult to avoid. The likelihood of such over-correction likely depends, however, on the decision processes triggered by each form of accountability and the propensity of the decision-makers to over-correct or engage in discrimination against the historically-advantaged groups within a market.

Relative to IC accountability, IB accountability should lead to more rigorous decision-making strategies by encouraging individuals to remain conscious of potential biases and to base their decisions on job-relevant characteristics [24]. IC accountability, in contrast, should lead to less rigorous decision-making strategies: the emphasis on group outcomes should simplify employment decisions by giving decision makers normative permission to resolve close calls in favor of traditionally disadvantaged groups and should encourage the use of simple counting heuristics in selection decision-making.

These considerations led us to predict that both IB and IC accountability should reduce the expression of bias against women and minorities, but they should achieve this positive result through decision-making strategies and normative pressures that lead to different risks of counter-bias against males and whites. In particular, IB is more likely to correct traditional bias without triggering a mirror-image bias in the opposite direction because it focuses attention on carefully weighing individual-level qualifications; in contrast, IC accountability is more likely to trigger over-correction of traditional biases, especially in labor pools in which traditionally disadvantaged groups have human-capital deficits, because discretion can now be used to give greater weight to criteria that favor members of the groups who are the focus of the organization’s diversification goals (i.e., screeners with discretion to determine the weight to give to different selection criteria will be able to construct a justification for their selection of the favored groups) [35].

*Hypothesis 1a*: *Both IB and IC accountability will reduce bias against women and minorities*, *but IC accountability will lead to bias against white males in applicant pools where women or minorities are relatively less qualified than white males*.

Additionally, the different decision strategies associated with IB and IC accountability should affect the quality of candidates selected. Relative to IC accountability, IB accountability should motivate more in-depth processing, leading decision makers to more carefully weigh job-relevant characteristics and to select more objectively qualified candidates. IC accountability will check bias by shifting evidentiary thresholds for preferring applicants with different category membership, resulting in the selection of less objectively qualified candidates.

*Hypothesis 1b*: *IB and IC accountability will produce differences in the quality of selected candidates*, *with IB accountability producing more qualified candidates than IC accountability*.

### Organizational Consequences of Accountability-Promoting Policies

Accountability pressures–particularly strong forms of outcome accountability–may also influence how decision makers feel about their company. Schoorman, Mayer and Davis [50] argue that control systems, including accountability policies, are an effective means of managing organizational risk, but they cautioned that control systems perceived to be too strong can impair trust between organizations and employees by limiting employee decisions and creating a situation where trustworthy behavior could be attributed to the controls rather than to the employee. In other words, stronger top-down controls over employee behavior signal to employees that they have less freedom in organizational processes and are less trusted by the organization’s executives. Because IC accountability limits managerial discretion to a greater extent than IB accountability, personnel decision-makers are likely to perceive IC accountability as implying that they are incapable of, or unwilling to make, fair hiring decisions, thus eroding trust (e.g., [51–53]). IB accountability, on the other hand, assigns to individual employees responsibility to act fairly in personnel matters and thus should convey a greater sense of trust by the organization.

*Hypothesis 2*: *IC accountability will lead to lower feelings of trust among those making selection decisions than IB accountability*.

As Bartlett [51] notes, when accountability is seen as coercion it may signal distrust and undermine support for the very goals the organizational seeks to achieve though the accountability system: “Social science research suggests that people are most likely to internalize norms when they feel autonomous, competent, and related to others. Coercion threatens these core prerequisites of the internalization process because it leads people to feel controlled, untrusted, and alienated” (p. 1902). Thus, whether IC accountability signals distrust is an important question for organizations that seek to use this form of accountability to advance diversity. If it does potentially send this message, then added steps may be needed to explain why this form of accountability has been chosen and to avoid employee alienation and resistance.

## Materials and Method

### Ethics Statement

The Institutional Review Board (IRB) of the University of California, Berkeley approved this research, and all participants were informed of their rights and of the risks of participation before proceeding to the experimental portion of the study. Before proceeding, participants gave their consent in writing pursuant to a consent procedure approved by the IRB.

### Participants

Two hundred and ninety-seven undergraduate business students participated in exchange for $15 (U.S.); data for eight participants could not be used because these participants failed to complete the experimental task as instructed. Two participants declined to report their sex and race; of the remaining 287 participants who provided usable data, 84 were male (30%), 203 were female (71%), 70 were white (24%), and 186 were Asian (65%). Although our participant sample was majority female, given that human resource departments often perform applicant screening functions in organizations and given that human resource departments tend to be female dominated (e.g., [54]), this skew in our sample arguably adds to rather than detracts from the external validity of our results.

### Procedure

This section summarizes the procedure used in the study. All study materials, including the participant consent form, the standardized instructions given to participants, the stimuli used and information about the pre-testing of the stimuli, the instruments completed by the participants, and the collected data, are publicly available at osf.io/6xqzh.

The experimental materials were presented via computer terminal. Participants read instructions asking them to assume the role of a human resources manager who must screen applicants for an entry-level managerial position at a regional office of a large company. Each participant received standardized summaries of applications that permitted ease of review of applicant information, ease of navigation and comparison of applicants, and ease of sorting applications into different evaluative categories. Each applicant summary provided the following information: (1) applicant name and photograph (names and photographs were pre-screened to be perceived as equally common and desirable and photographs were pre-screened to be perceived as comparable in age, socio-economic class, attractiveness, and professional appearance and to be seen as clearly black or white and male or female); (2) number of years of relevant work experience, ranging from three to six years (experience level was randomly assigned to applicants for each participant); (3) a score on a technical-skill test (“excellent,” “very good,” “good,” “average,” “poor,” “very poor” or “unsatisfactory”); and (4) a score on a teamwork-skill test (“excellent,” “very good,” “good,” “average,” “poor,” “very poor” or “unsatisfactory”).

Photographs were added to the applications to ensure that race and gender differences across candidates were apparent. Manipulating physical appearance is a more reliable means of manipulating the race and gender of applicants than manipulating applicant names or resume content (i.e., participants may not share common beliefs about the gendered or ethnic nature of different names or different resume content) [55]. Participants did not voice any suspicions about the presence of applicant photographs.

With respect to applicant names, a long list of applicant names was compiled from a database of popular baby names. Six male and female research assistants then rated each name for its perceived gender, commonality, desirability, and for whether it was associated with any particular racial or socio-economic group. This process was used iteratively to develop a set of comparable names by perceived gender. A similar process was used for applicant photographs, with the research assistants being asked about the perceived age, gender, race, class, and attractiveness of the person in each photograph. (These pretesting materials are archived at osf.io/6xqzh.)

Participants were presented with 75 application summaries that comprised the full applicant pool. This screening process was fashioned after the process used by many organizations with whom the authors have consulted that receive multiple applications for a limited numbers of openings, particularly with the rise on online recruiting and applications [56]. To deal with the multitude of applications, many organizations employ a multi-step screening process, the first step of which involves sorting between applicants who do and do not meet minimum job requirements and then sorting among those who do meet minimum requirements to decide who should receive an interview or not. Many organizations have moved to applicant tracking systems to systematize and streamline the applicant screening process [57].

Using a Q-sort methodology [58], participants were instructed to sort the applicants into seven categories for purposes of interview selections: (1) “definitely interview” (eight candidates were to be placed in this category), (2) “very likely to interview” (ten candidates), (3) “likely to interview” (12 candidates), (4) “possibly interview” (15 candidates), (5) “unlikely to interview” (12 candidates), (6) “very unlikely to interview” (ten candidates), and (7) “definitely not interview” (eight candidates). Each applicant was assigned a score based on the interview category into which each was placed, ranging from 1 (best) to 7 (worst).

A subset of 36 applications was formed using a within-subjects factorial design that crossed Applicant Sex (male or female), Applicant Race (black or white), Technical Skill (good, average or poor) and Teamwork Skill (good, average or poor). This set of applications was used to examine the impact of sex, race and qualifications on interview ratings across all experimental conditions. The composition of the remaining 39 applications presented to participants varied by labor-pool condition, as described below; this subset of applicants was used to examine how labor pool conditions affected the impact of candidate qualifications and demographics on interview ratings.

Participants were told that both technical skill and teamwork were important for the open positions, but participants were not instructed on how much weight to give to these factors. Rather, they were told that “your boss is leaving it up to you to decide how much weight to give” to each candidate’s qualifications. This approach was taken to ensure subjective judgment in the review process, because subjective judgment is hypothesized to be the primary avenue through which intergroup bias enters into the personnel selection process (see, e.g., [33, 34, 59]). Furthermore, because participants could give a large number of candidates positive ratings (45 candidates could be placed into categories ranging from definitely interview to possibly interview) rather than making a small number of yes/no interview decisions, the IC accountability manipulation did not function as a hard constraint on selections. Any bias that might appear would take the form of relatively less favorable ratings for one group versus another, differences that could be easily justified given participant discretion in how to weigh candidate qualifications.

After sorting applications, participants answered five questions (α = .59) measuring how much they felt the company trusted them to make personnel decisions (responses on a 7-point scale: 1 = strongly disagree and 7 = strongly agree): (1) “I felt trusted to select the best candidate for the position;” (2) “I felt overly constrained in choosing between the candidates” (reverse coded); (3) “Management did not trust me to review the candidates fairly” (reverse coded); (4) “I was not given enough credit that I would fairly choose between candidates” (reverse coded); (5) “Management would support any interview decision I made.” Participants were then debriefed and paid for their participation.

#### Accountability manipulation

Participants were randomly assigned by computer program to one of three, between-subjects, accountability conditions. Participants in the no-accountability condition were told that “[y]our judgments will be completely anonymous and in no way traceable to you.” Participants assigned to the IB accountability condition were told that they would have to explain in detail the process by which they made their decisions to an experimenter “who suspects that discrimination remains a serious problem in workplaces and who will be checking to ensure that each candidate was judged consistently and fairly and that the principles of equal employment opportunity are respected.” (No experimental session involved more than two participants so that participants would believe that the experimenter would have time for a one-on-one discussion with each participant.) Participants in the IC accountability condition were told that they would have to justify their decisions to an experimenter “who suspects that discrimination remains a serious problem in workplaces and who will be comparing the ratio of women and minorities to be interviewed to the female and minority applicants in the overall labor pool–in this case 60%. The experimenter will scrutinize decisions especially closely when the decisions produce fewer female and minority interviews than the target goal number.” The IC accountability manipulation thus utilizes targets rather than quotas, as companies typically use aspirational goals rather than hard quotas when implementing identity-conscious affirmative action plans. Quotas that explicitly make candidate race or sex a deciding factor raise legal concerns [60]. Affirmative action plans have become common among large employers. For example, Kalev et al. [5] found that, by 2002, 63% of the 708 firms they studied had implemented such a plan. Such plans often specify that special efforts during recruitment, hiring and promotion should be made to increase the representation of women and African Americans within a company but, as with our manipulation, do not specify that group identity should trump qualifications.

### Labor-pool manipulation

Following the procedure used in Mellers and Hartka [61], the aggregate qualifications of male versus female applicants and white versus black applicants were manipulated to examine how differences in the composition of the labor pool affected screening decisions. In particular, participants were randomly assigned to one of two, between-subjects, labor-pool conditions. In the *equal-advantage condition*, applicants from the four target demographic groups (white males, white females, black males, and black females) were assigned technical-skill and teamwork-skill scores so that each sex and race had equal scores on average (correlation of 0 between sex/race and technical/teamwork-skill score). Thus, in this condition, none of the demographic groups possessed a human capital advantage within the applicant pool. In the *majority-advantage condition*, applicants were assigned technical-skill and teamwork-skill scores so that the male candidates and the white candidates had higher scores on average than did female candidates and black candidates (correlation of .21 between sex/race and technical/teamwork-skill scores). Thus, in this condition, male candidates and white candidates possessed a human capital advantage in the applicant pool.

## Results

We first examined whether participants made meaningful distinctions among applicants based on applicant qualifications. Across all experimental conditions, analysis of variance revealed that candidates with higher technical-skill scores and higher teamwork-skill scores received higher interview scores than less qualified candidates using Fisher’s least significant differences method as a correction for multiple comparisons (*p*< .01 for every combination). (Whenever multiple comparisons were conducted, Fisher’s least significant difference method was used to correct for the possible inflation of the Type I error rate.) This pattern indicates that participants attended to the teamwork and technical skill information and treated it as job-relevant information with some predictive utility (for a discussion of the benefits and limits of performance ratings for predicting future work, see [62, 63].

Second, we examined whether participants exhibited bias against any group of applicants. In the equal-advantage labor pool, participants preferred female candidates over male candidates (mean interview rating = 4.00, sd = .02 versus mean rating = 4.05, sd = .01; *F*(1,30) = 5.85, *p* = .02, η^2^ = .16).

Although there was no significant main effect of race in the equal-advantage labor pool, there was a significant three-way interaction among applicant race, technical skill, and teamwork skill (*F*(4,120) = 3.73, *p* < .01, η^2^ = .11). Black candidates with good technical-skill scores but poor teamwork scores (or with poor teamwork scores but good technical-skill scores) received better ratings than white candidates with the same objective qualifications. This strategy suggests that black applicants received more “benefit of the doubt” when there was greater ambiguity about their skills (cf. [35]).

In the majority-advantage labor pool, female candidates received higher mean interview ratings than equally-qualified males, but this difference did not pass the traditional threshold for statistical significance (mean interview ratings for females and males were 3.98 (sd = .02) and 4.03 (sd = .01), respectively, *F*(1,31) = 3.83, *p* = .06, η^2^ = .11). There were no significant effects due to race (*F*(1,31) = .48, *p* = .49) in this pool.

### Hypothesis 1: The Impact of Accountability

Hypothesis 1a stated that both IB and IC accountability would reduce anti-female and anti-minority bias but that IC accountability would be more likely to trigger a counter-bias against men and whites. In the IB accountability condition, there were no significant differences in the treatment of each minority group relative to equally-qualified white males in either of the two labor pools. All six comparisons did not differ significantly from 50%. However, in the IC accountability conditions, all three groups of minority candidates in the equal-advantage labor pool and two of the three minority-group candidates in the majority-advantage labor pool were significantly more likely to be selected over equally-qualified white males. The chances that the higher ratings of minority candidates happened on the basis of chance were, respectively, .04, .0005, and .003 for white females, black females, and black males in the equal-advantage pool and .002, .002, and .07 for the same three groups in the majority-advantage pool. Probabilities are based a binomial distribution. Consistent with Hypothesis 1a, IB accountability greatly reduced inequalities across groups without triggering a counter-bias against white males, whereas IC accountability triggered this bias.

**Fig 1:**
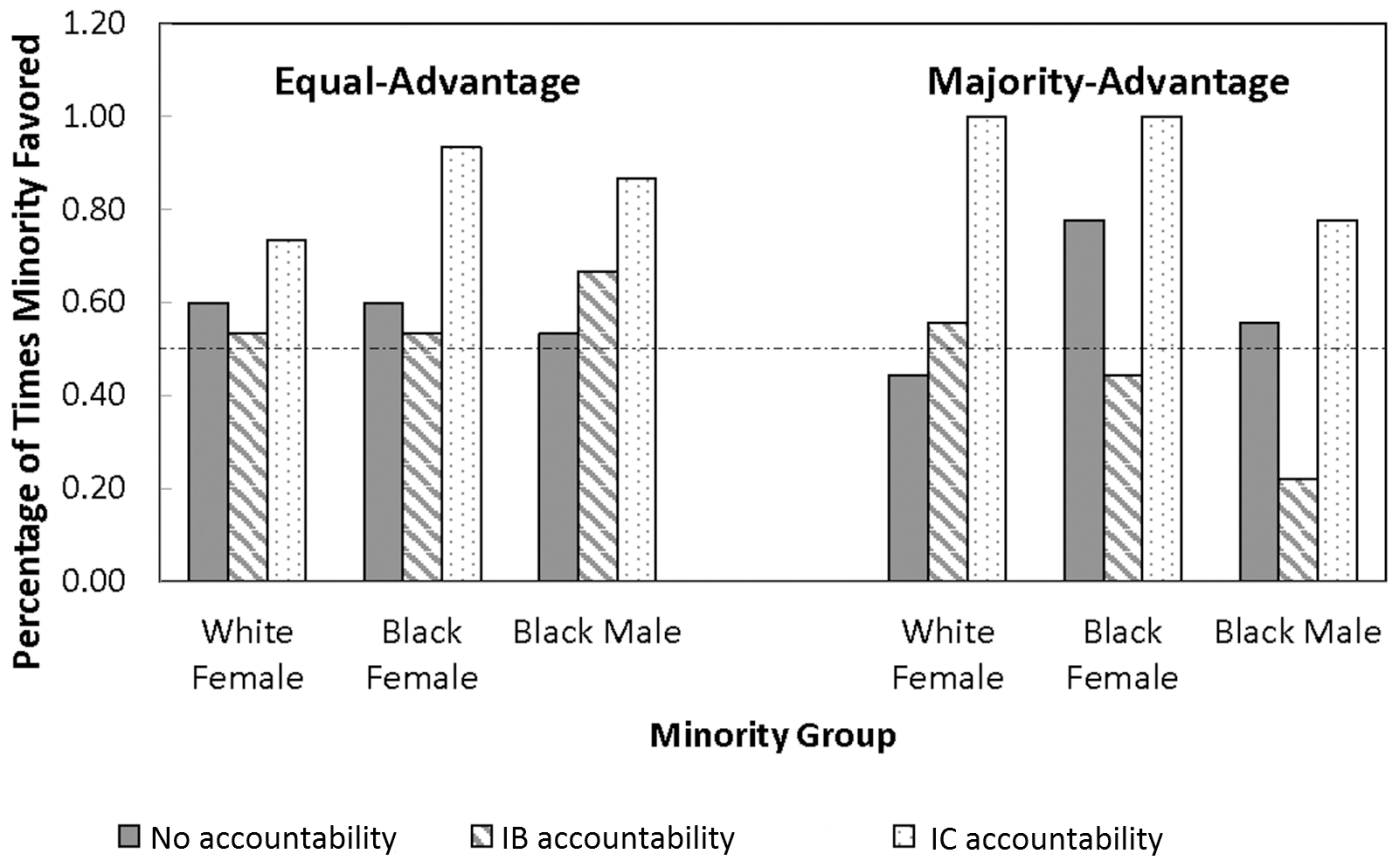
Frequency of Pro-minority Advantage by Labor Pool.

**Fig 2:**
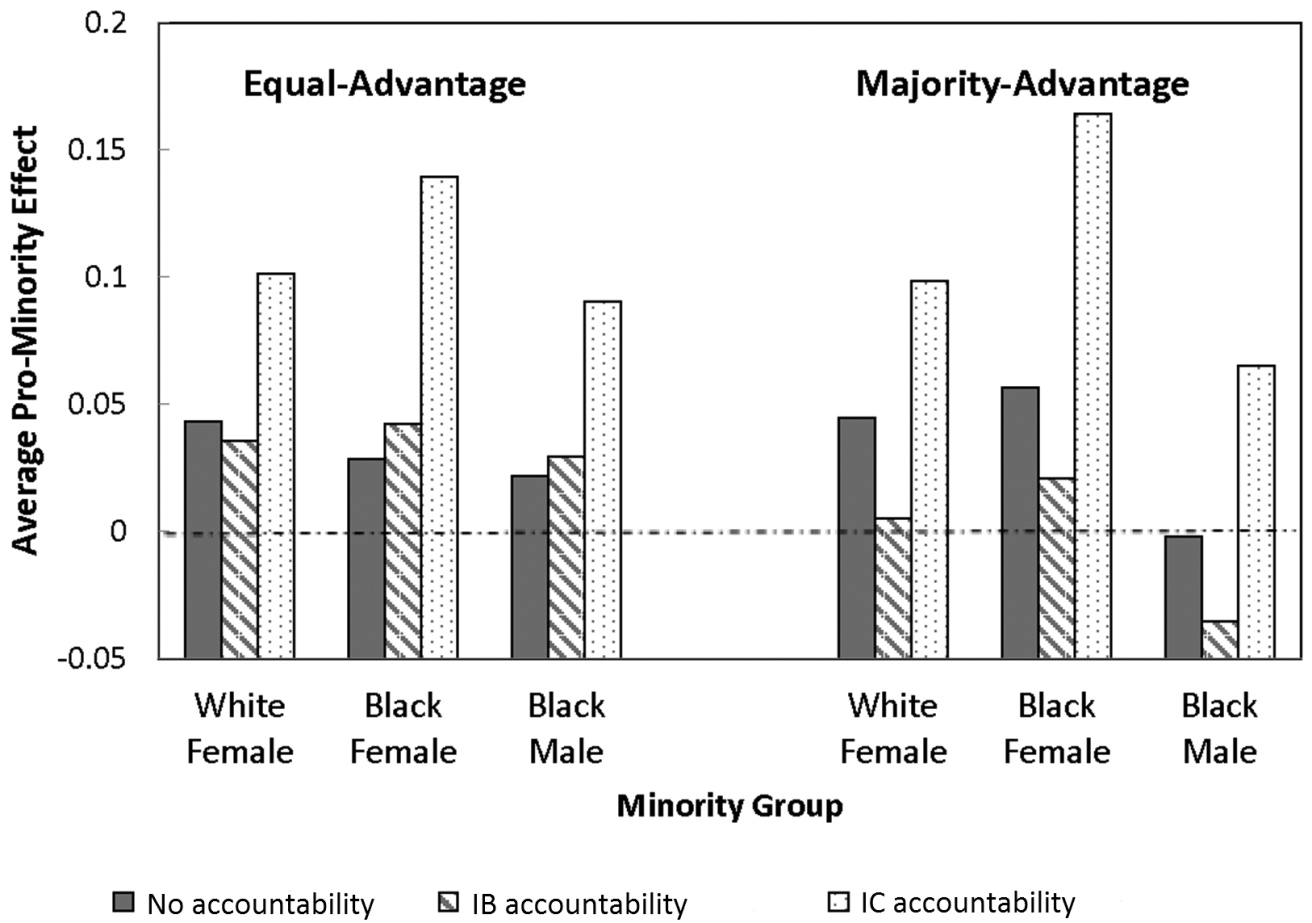
Size of Pro-minority Advantage by Labor Pool.

Figs 1 and 2 show the differential treatment of the minority groups relative to white males under the different accountability and labor pool conditions. Fig 1 presents the percentage of times a minority candidate was preferred to equally-qualified white males out of fifteen comparisons in the equal-advantage labor pool and nine comparisons in the majority-advantage labor pool. If minority and majority group candidates were treated equally, percentages of pro-minority ratings would be 50%. Under IB accountability, minority and majority groups were treated equally in either of the two labor pools. None of the six comparisons differed from 50%. However, under IC accountability, all three groups of minority candidates in the equal-advantage labor pool and two of the three minority-group candidates in the majority-advantage labor pool were significantly more likely to be selected over equally-qualified white males. Binomial probabilities were .04, .0005, and .003 for white females, black females, and black males in the equal-advantage pool and .002, .002, and .07 for the same three groups in the majority-advantage pool.

Fig 2 presents average differences in the size of the pro-minority advantage relative to white males. Under IB accountability, there were no significant differences in either labor pool between interview ratings of each of the three minority groups relative to equally-qualified white males. However, under IC accountability, all three minority groups received higher average ratings than white males in the equal-advantage labor pool (*t*(14) = 4.31, 4.73, and 3.25 for black females, black males, and white females, respectively, all *p*’s < .01) and in the majority-advantage labor pool (*t*(8) = 10.35, 2.16, and 4.51 for black females, black males, and white females, respectively, all *p*’s < .01). Consistent with Hypothesis 1a, IB accountability greatly reduced inequalities without triggering a mirror-image bias against white males, whereas IC accountability triggered over-correction.

We found that a pro-female bias emerged under IC but not IB accountability; however, because there were more female than male participants in our sample, it is possible that an in-group bias (with female participants preferring female candidates) rather than IC accountability pressure accounted for this result. To test this possibility, we predicted interview ratings from participant sex, candidate sex, and the interaction of these two variables, controlling for accountability condition, labor pool condition, candidate race, and candidate technical-skill and teamwork-skill scores. Neither participant sex (*t*(1) = .45, *p* = .73) nor the interaction between participant sex and candidate sex (*t*(3) = -1.14, *p* = .33) significantly predicted placement of candidates into interview categories, contrary to an in-group-bias explanation for the pro-female bias found under conditions of IC accountability.

Hypothesis 1b posited that accountability would have consequences for the quality of candidates selected by participants. In particular, IB accountability was expected to induce more in-depth processing and result in the selection of better-qualified candidates, whereas IC accountability was predicted to shift attention toward demographic characteristics and away from job-relevant qualifications. We tested this hypothesis by computing the average ability score for each candidate in each interview category (because we did not specify that either score was more important than the other, we treated each skill as equally important, with higher scores representing higher rated ability) and examining whether these differences were statistically significant using t-tests. Comparisons were made between IC and IB accountability conditions within a given labor pool (in which qualifications of minorities and majorities were held constant) but not across labor pools. Support for Hypothesis 1b would be found in higher average ability scores in the top interview categories (1, 2, and 3), and lower average ability scores in worst interview categories (5, 6, 7) under conditions of IB accountability. That is, Hypothesis 1b posits that, relative to IC accountability, IB accountability will result in higher-qualified candidates being more likely to be interviewed and less-qualified candidates less likely to be interviewed. Table 1 provides information on the average combined skill set possessed by candidates placed into the interview rating categories across the combination of labor market and accountability conditions (higher averages reflect candidates with better scores on the measures of teamwork and technical skill).

**Table 1:** Mean Combined Teamwork and Technical Skill Scores of Applicants by Interview Category and Experimental Condition.

		Equal Advantage Labor Pool					Majority Advantage Labor Pool				
Interview Category	Account-ability	Mean Skill	SD	t-stat	df	*p*	Mean Skill	SD	t-stat	df	*p*
**1**	**IB**	5.49	.51	.51	494	.61	5.92	.69	-.03	518	.98
	**IC**	5.47	.52				5.92	.71			
**2**	**IB**	4.97	.14	3.49	618	.001	5.01	.27	2.18	648	.03
	**IC**	4.92	.23				4.95	.34			
**3**	**IB**	4.49	.26	-.82	742	.41	4.50	.32	-1.42	778	.16
	**IC**	4.51	.32				4.53	.31			
**4**	**IB**	3.98	.24	-.37	928	.71	3.97	.25	-.11	973	.91
	**IC**	3.98	.27				3.97	.25			
**5**	**IB**	3.51	.26	1.16	742	.25	3.51	.36	-1.20	778	.23
	**IC**	3.49	.31				3.54	.33			
**6**	**IB**	3.06	.23	-2.83	618	.005	3.00	.37	-.21	648	.83
	**IC**	3.13	.39				3.01	.32			
**7**	**IB**	2.51	.52	.08	494	.94	2.12	.68	.64	518	.52
	**IC**	2.51	.50				2.08	.67			

In the experiment, participants placed applicants into an interview category ranging from 1 (definitely interview) to 7 (definitely not interview). This table presents comparisons of the mean combined teamwork and technical skill scores for applicants (higher means signify better qualifications) by interview category under conditions of identity-blind (IB) versus identity-conscious (IC) accountability across the two labor pool conditions (in the equal advantage pool, white male applicants were no more qualified on average than other applicants; in the majority advantage pool, white males did have a qualifications advantage). Different degrees of freedom across the interview categories reflects the fact that participants were forced to place fewer applicants into the high and low categories.

In several interview categories, there were no significant changes in qualities of candidates due to accountability condition. However, when significant differences in abilities emerged, results were consistent with Hypothesis 1b. When majority and minority candidates had the same advantages in the labor pool, IB accountability resulted in better-qualified candidates being placed in interview category 2 (*t*(618) = 3.49, *p* < .01) and worse-qualified candidates being placed in interview category 6 (*t*(618) = -2.83, *p* < .01), as compared to the placements made under IC accountability. When majority candidates had the advantage in the labor pool, IB accountability led to better-qualified candidates being placed in interview category 2 relative to placements under IC accountability (*t*(648) = 2.18, *p* = .01). In sum, participants held accountable for being identity blind (versus identity conscious) gave better-qualified candidates a greater chance of being interviewed and worse-qualified candidates a lower chance of being interviewed.

### Hypothesis 2: Accountability and Trust

Hypothesis 2 stated that IC accountability, a form of outcome accountability aimed at restricting employee autonomy, would lower the sense of being trusted among those making selections. Consistent with Hypothesis 2, analysis of variance revealed that participants felt less trusted under IC accountability (mean = 4.76, sd = .10) than under no accountability (mean = 5.06, sd = .10; *F*(1,123) = 4.28, *p* = .04, η^2^ = .03). This result held true in the majority-advantage labor pool (*F*(1,123) = 3.97, *p* = .048, η^2^ = .03) but not in the equal-advantage labor pool (*F*(1,123) = .91, *p* = .34). Trust levels under IB accountability (mean = 4.99, sd = .10) did not differ from those under no accountability (mean = 5.06, sd = .10; overall *F*(1,122) = .23, *p* = .63), regardless of whether participants considered the equal-advantage labor pool (*F*(1,122) = .00*p* = 1.00) or the majority-advantage labor pool (*F*(1,122) = .45,*p* = .50).

## Discussion

These findings shed new light on the risks and benefits of alternative forms of selection accountability and, more broadly, on different philosophical approaches to ensuring equal-employment opportunity and social justice [64, 65, 66]. First, both IB and IC accountability prevented discrimination against women and African-American applicants, but IC accountability resulted in a bias against white male applicants. This bias became more pronounced in the experimental condition in which traditionally disadvantaged minority groups suffered from human-capital deficits, which is precisely the kind of labor market in which companies are likely to employ strong forms of affirmative action to redress historical inequities. Despite the simulated nature of our human resource task and the fairly mild form of IC accountability imposed on participants, we observed effect sizes that were equal to or larger than most effects observed within social psychology, including other studies of racial and gender bias [67, 68]. In real organizational settings, managers who have control over resources that are important to subordinates may impose more powerful forms of IC accountability, with possibly even larger effects.

Second, the pro-female and pro-black biases observed under IC accountability conditions resulted in the selection of less-qualified candidates across labor pools. These results are consistent with research suggesting that accountability instructions that focus decision-makers on outcomes, as opposed to processes, result in more cursory information processing and a shift in attention from job-relevant to job-irrelevant factors.

Third, participants reacted negatively to IC accountability instructions, reporting feelings of less trust in their decision-making ability. These results suggest that strong forms of outcome accountability may impose a social cost on the organization and have negative implications for the psychological contract between managers and the organization. Our study was designed to examine the basic relation of accountability and trust rather than to identify the nature of that relation. It may be that outcome-accountability pressure is aversive because it signals a negative organizational view of the decision-maker or because it violates meritocracy-grounded views of informational and interactional justice [69]. Future studies will be needed to gain a better understanding of the source of the perceived distrust.

These results demonstrate that, for organizations seeking to debias their personnel selection processes rather than achieve hiring targets, IB accountability is likely to provide a more effective intervention than IC accountability. Regardless of the composition of the applicant pool, the IB accountability instruction resulted in better calibrated bias mitigation, reducing bias without prompting over-correction and yielding a better-qualified pool of interviewees.

Finally, our results have important implications for the study of accountability. As we discussed in our Introduction, researchers have paid relatively little attention to the different psychological and behavioral effects of alternative forms of accountability. Our results show that focusing those who are accountable on the outcomes of a process can have very different effects than focusing those who are accountable on following a desired procedure. This distinction between process and outcome accountability transcends the human resource domain that was our concern and deserves further study in this and other domains.

## Limitations and Future Directions

Although this study advances our understanding of how different accountability interventions affect bias in personnel selection, the study’s limitations should be considered. First, we utilize a student population rather than human resource professionals, hiring managers, or other people with experience making the sort of interview-triage decisions our participants were asked to make. Furthermore, in some settings those who conduct applicant screening will have more knowledge about job duties and will have more information about the candidates than our participants did, conditions that may make the screeners more confident in their judgments and less susceptible to pressure from managers to give preference to any particular group. Future researchers may wish to replicate our experimental paradigm using such working populations in order to understand whether expertise or experience with the task alters the results. We predict that the pattern of results would be robust to participant populations given the clarity of our results, but the magnitude and direction of bias is likely to change with organizational context, legal climate, and applicant mix. Likewise, accountability research suggests a high level of generalizability in the ways in which people are influenced by accountability pressure, making it unlikely that a student sample would be affected substantially differently by the study conditions.

Second, we recognize that it is possible to agree about the need to study the consequences of debiasing interventions but still have concerns about the particular laboratory experiment presented here. Critics could argue, for instance, that although the current research paradigm may satisfy the logical requirements for testing for gender and racial bias, it does not satisfy the psychological requirements. In this vein, they could maintain that it is too easy for participants in repeated-measures designs of the current sort to figure out which hypotheses are being tested–and to position themselves in a socially desirable light. According to this logic, participants in our control condition were only fair-minded because many were already predisposed to be egalitarian and virtually all were aware of ambient egalitarian institutional norms and aware they were making judgments under a behavioral-science microscope. In this view, we need to create a tougher series of tests that draw on more representative samples of participants, that rely on between-subjects rather than repeated-measures designs, and perhaps that give participants an opportunity, prior to making simulated personnel decisions, to demonstrate their "moral credentials" [70], thereby further reducing the level of evaluation apprehension arguably present even in control conditions.

Unfortunately for managers seeking guidance from simulation research, there is no objectively right answer to the question of how difficult it should be to “pass” laboratory tests for bias or organizational training exercises. One can defend the current paradigm and results on mundane-realism grounds by arguing that: (1) personnel decision-makers in large companies live in a largely repeated-measures world similar to that of the current study where they focus their attention on a set of particular job-relevant factors measured in more and less objective ways; (2) given the large number of candidates who differed along multiple qualification and group-identity dimensions, no single set of normatively correct selections was apparent; (3) personnel decision-makers operate in a legal environment in which it is illegal to discriminate and the accountability stakes for discriminators are certainly higher than those in our experiment; (4) personnel decision-makers typically go about their work without previously being exposed to moral-credentialing manipulations; (5) our sample was predominantly female, but human resource departments are as well. The only conclusion we can draw here with reasonable certainty is that no single paradigm will suffice for the multifaceted challenge of assessing the degree to which subjective personnel decision-making is untainted by factors outside of legitimate, job-related qualifications.

## Conclusion

The practical import of the current findings is the need to calibrate debiasing manipulations to the types of personnel decisions that managers are making and to the types of populations from which both managers and employees are drawn. If companies are operating in a labor pool in which there are large human-capital deficits in traditionally disadvantaged communities and if the companies’ managers display little or no baseline bias against traditionally disadvantaged groups (or, equivalently, if the company has a positive record of diversity and inclusion), then companies that implement identity-conscious forms of selection accountability may pay a potentially high price in terms of workforce qualifications and efficiency–as well as a price in employee trust. Conversely, a company that relies on identity-blind selection accountability should reduce the odds of discrimination by its managers, increase the validity of its personnel selections, and increase managerial trust, but at this company the representation levels of women and minorities will depend on how well these groups compete with white males in the applicant pool. Our results suggest that companies that seek to avoid bias against all groups, select the best candidates, and have a diverse workforce should combine identity-blind selection accountability with affirmative efforts to ensure high levels of well-qualified women and minorities in the applicant pool.
